# A Longitudinal Examination of Withholding All or Part of School Recess on Children’s Physical Activity and Sedentary Behavior: Evidence from a Natural Experiment

**DOI:** 10.1007/s10643-022-01325-2

**Published:** 2022-02-24

**Authors:** Alejandro Carriedo, José A. Cecchini

**Affiliations:** 1 Department of Education Sciences, University of Oviedo, Oviedo, Spain; 2 Department of Education Sciences, Faculty of Teacher Training and Education, University of Oviedo, C/ Aniceto Sela, s/n, Office 215, Oviedo, Spain

**Keywords:** Children’s health, Children’s behavior, Playground, School policies, Teacher’s behavior

## Abstract

School recess is a daily opportunity for school-age students to be physically active. However, in some territories teachers often use recess for other purposes (e.g., children’s poor classroom behavior might be punished with reduced time for recess). This study aimed to examine the impact of such practices on children’s physical activity (PA) and the relationships between PA, gender, body mass index (BMI), and academic achievement. Forty-six first-grade students from two natural classrooms wore an accelerometer over the course of 6 weeks to measure their metabolic equivalent of task (METs) and sedentary behavior during school recess. Gender, age, BMI, the classroom to which students belonged, and academic achievement were also analyzed in two Generalized Estimating Equations models. Results revealed that boys achieved more METs and spent less time participating in sedentary behavior than girls during recess. Children within a healthy weight range of BMI yielded more METs than underweight and overweight/obese children. Academic achievement was positively associated with the METS and negatively with the sedentary behavior. Finally, withholding all or part of school recess significantly reduced children’s PA and extended their sedentary behavior. The literature indicates that school recess plays an important role in promoting numerous children’s health outcomes. Therefore, students should not be excluded from participation in all or part of recess.

## Introduction

The benefits of regular participation in physical activity (PA) in childhood have been extensively investigated, showing inverse relationships between PA and cardiovascular risk, and with beneficial effects on several mental health outcomes (Bull et al., 2020; Penedo & Dahn, 2005). Likewise, PA has proved to be an important factor in obesity and disease prevention in children (Janssen & LeBlanc, 2010; Strong et al., 2005). There is also evidence suggesting that PA is associated with several aspects of brain function and cognition (Ellemberg & St-Louis-Deschênes, 2010; Fedewa & Ahn, 2011; Hillman et al., 2008). Thus, both acute and chronic moderate to vigorous PA interventions might produce changes in brain structure and function in children aged 6–12 years, as well as cognition, and academic outcomes (Erickson et al., 2019, Greef et al., 2018). These changes may improve cognitive functions such as concentration, attention, executive function, and working memory (Donnelly et al., 2016; Hillman et al., 2008) which are important for academic success (Fedewa & Ahn, 2011). For instance, several controlled studies have confirmed that PA can enhance academic content learning such as language (Barnett et al., 2008) or mathematics (e.g., Cecchini & Carriedo, 2020). Consequently, improvements in these cognitive functions as a result of increased PA might, in turn, improve children’s academic achievement (Gonzalez-Sicilia et al., 2019; Mullender-Wijnsma et al., 2016; Zeng et al., 2017). In this regard, the relationships between PA and physical and cognitive health indicators are more consistent and robust for higher versus lower intensity PA (Poitras et al., 2016).

PA intensity can be classified by rate of energy expenditure through a continuous indicator known as metabolic equivalent of task (MET). The MET is a system used to calculate the energy requirements for PA. One MET corresponds to the equivalent of the energy needed for the basal metabolic rate. It is commonly established that light PA (e.g., casual walking, stretching) requires less than 3 METs, moderate PA (e.g., playground games, dancing) requires between 3 and 5.9 METs, and vigorous PA (e.g., soccer, swimming) requires more than 6 METs (Ainsworth et al., 2011). World Health Organization (Bull et al., 2020) recommends that children accumulate an average of 60 min/day of moderate to vigorous PA (MVPA) across the week. However, some studies have shown that the majority of school-age children fail to meet these recommendations (Cooper et al., 2015; Zimmo et al., 2017), which represents a serious concern in public health. Furthermore, after the declaration of the COVID-19 global pandemic, most governments have imposed several physical distancing measures and contact restrictions to fight the sharp rise in coronavirus infections (Carriedo et al., 2020). These protective measures have resulted in a radical change in the lifestyle of the population and could have reduced the PA patterns in children, leading to increased risk of obesity, diabetes, and cardiovascular disease (Dunton et al., 2020).

On the other hand, sedentary behavior, defined as “any waking behavior characterized by an energy expenditure ≤ 1.5 METs while in a sitting or reclining posture” (Sedentary Behavior Research Network, 2012), is an independent construct with different potential health consequences (Lopes et al., 2017). It has been reported that large amounts of sedentary time prolonged might contribute to increase body mass index (BMI) and fatness (Cliff et al., 2014; Marques et al., 2016; Zhang et al., 2016), and it is one of the most important predictors of morbidity and mortality risk (Biddle et al., 2016; World Health Organization, 2004). In this regard, it should be noted that obese children participate in significantly less daily MVPA than healthy weight children (Page et al., 2005) and that normal or healthy BMI has been positively associated with the intensity of PA during school recess (Ridgers et al., 2014). Although causality in this relationship remains unclear (Greca, 2017), it is highly recommended to break up long periods of sitting and avoid sedentary time as frequently as possible during the day (Carson et al., 2016; Júdice et al., 2017). Several studies indicate that sedentary behavior contributes to overweight, that children with obesity keep their unhealthy weight, and that healthy weight children might be more likely to become overweight (Page et al., 2005; Tremblay et al., 2011).

Elementary school children, who spend a considerable part of the day at school, are predominantly seated during traditional lessons, between 76 and 97% of the time (Cardon et al., 2004; Mooses et al., 2017). Therefore, school recess is not only a necessary break from the rigors of academic tasks in the classroom or a period where students have an opportunity to freely experience socialization and communication but it also plays a crucial role in contributing to the children’s PA levels and subsequent health and cognitive benefits (Ramstetter et al., 2010). School and specifically physical education lessons have been widely examined to identify (Júdice et al., 2017; Mooses et al., 2017) or enhance (Graham et al., 2014) PA levels and sedentary behavior in children. However, recess symbolizes a daily free play opportunity and children should be encouraged, but not required, to be physically active during playtime (Ramstetter et al., 2010), which might contribute to 5–40% of recommended daily PA levels when no interventions have been utilized (Ridgers et al., 2006).

It has been detected that in some countries the pressure to improve academic performance often leads to allocating more instructional time for subjects such as language and mathematics; likewise, other school policies allow teachers to withhold all or part of the recess to punish children’s misbehavior or to finish classwork (Ramstetter et al., 2010). Together, these practices might cause some students to not reach the recommended PA levels for health (Bull et al., 2020). Hence, to understand PA patterns in school-age students it is important to know how different school policies [e.g., using recess for other purposes such as to punish children’s misbehavior or to finish classwork (Ramstetter et al., 2010)] might impact on the student’s PA levels.

Therefore, considering this background, five objectives were proposed: (1) assess first-grade students’ PA levels and sedentary behavior during school-recess; (2) examine the relationships between sedentary behavior and METs achieved during school recess with the academic achievement, day of the week, and weeks; (3) analyze gender differences; (4) examine the METs and sedentary time yielded by students during school recess according to their BMI; and finally, (5) examine whether the use of recess to work on other educational issues has a significant impact on the PA levels and sedentary behavior of first-grade students during school recess.

## Material and Methods

### Participants

Nineteen boys and 27 girls (*N* = 46) enrolled in two first-grade classrooms selected by convenience from one school located in northern Spain returned signed parental informed consent to participate in a 6-week longitudinal observational study. All were Caucasian. The ages ranged from 5 years and 10 months to 6 years and 10 months (*M* = 6 years and 5 months, *SD* = 4 months). Forty-eight children were invited to participate; however, two children did not return signed parental consent (4.16%). Thus, the equations related to finite populations lead to a 2.98% margin of error with 95% confidence level.

### Instruments and Measures

#### METs and Sedentary Behavior

ActiGraph-GT3X (ActiGraphTM, LLC, Fort Walton Beach, FL, 154 USA) activity monitors were used to provide estimates of energy expenditure (i.e., METs) and time spent in sedentary behavior (< 1.5 METs), light PA (1.5–3.99 METs), moderate PA (4–5.99 METs), vigorous PA (6–8.99 METs), and MVPA (≥ 4 METs) of children (Saint-Maurice et al., 2016) during school recess. Activity monitors were initialized to measure triaxial acceleration and to collect data in 10-s epochs. There is no consensus on the best cut-points for the children’s MVPA classification (Kim et al., 2012). In this study, Freedson et al. (2005) cut-points were used to determine children’s energy expenditure and PA intensity categories because they have demonstrated good agreement with measured PA intensity and good accuracy in classifying MVPA, specifically for children ages 6–10 years (Kim et al., 2012).

#### Other Measures

Other measures included in the analysis were age; gender (0 = boys, 1 = girls); group (0 = B, 1 = A); participant’s BMI [0 = underweight (< 5th percentile), 1 = normal or healthy weight (5th percentile to the 85th percentile), 2 = overweight/obese (> 85th percentile)] was calculated from the ratio weight/height^2^ (kg/m^2^) and percentiles were determined using the Center for Disease Control and Prevention (2015) criteria according to a child’s gender and age (in this sample, 6.5% were underweight, 78.3% were normal or healthy weight, 10.9% were overweight, and 4.3% were obese [> 95th percentile]. The physical education teacher measured the children’s stature and body mass using standardized procedures. For data analysis in this study, the overweight and obese group were merged to form one group termed overweight/obese (> 85th percentile); academic achievement [the average of the grades obtained in all core subjects (mathematics, language, natural sciences, social sciences, first foreign language), score range 1–10 points]; day of the week (1 = Monday, 2 = Tuesday, 3 = Wednesday, 4 = Thursday, 5 = Friday); and weeks (ordinal variable ranging from 1 to 6).

### Procedure

First, the research ethics committee of the University reviewed and approved this study, whose procedures followed were in accordance with the Declaration of Helsinki 1975, revised Hong Kong 1989. Then, the principal of the school authorized the study. Finally, informed consent was obtained from all participants’ parents.

All participants wore an adjustable belt containing an accelerometer around the non-dominant hip for 6 weeks that included 23 school days (from 9 a.m. to 2 p.m.). Verbal instructions were given by the researchers regarding how the accelerometer had to be worn and a demonstration was given. Participants followed their normal daily school routines, with PA being monitored during the complete school day. Recess time was determined as the time that school bell rang to start recess (12:00 p.m.) until the time it rang to conclude recess (12:30 p.m.). Thus, it was considered 30 min wear time as the criterion for a valid recess. During the 6 weeks, some students missed some days of class, resulting in 17.49% of missing data. All students participated in at least 20 recess periods.

A researcher visited the school every day for 6 weeks and tried not to interact with children and teachers. The visits formed part of a longitudinal study which was investigating the PA levels of first-grade children during recess time. Thus, the research design of this study (i.e., longitudinal study using a natural experiment) implied that the researchers did not interfere with students or teachers and several observations (i.e., 23 time points during 23 school days) of the PA levels were registered over 6 weeks. Thus, the data collected in this study could determine patterns efficiently and connections can be made in a clearer manner. However, participants belonged to two different classrooms (A group, *n* = 23; B group, *n* = 23) and they had two different teachers who used different methodological practices. Consequently, different patterns of PA were observed between the classrooms in the first week. In order to understand such dissimilarities, the researcher carefully observed the normal daily school routine of students and detected that all students from the “B” group were in the playground from almost the time the school bell rang (12:00) to the time it rang to conclude recess (12:30) whereas, students that belonged to the “A” group tended to stay in their classroom for an undetermined period of time when the school bell rang. Furthermore, students from the “A” group began to leave the classroom intermittently. That is to say, students left the classroom when (a) they finished the classwork, (b) when they eventually understood the content, (c) when the teacher lifted some type of punishment, or (d) when students freely decided to go to the playground. This pattern did not happen on Fridays because the teacher of the “A” group had to supervise students during recess time (i.e., playground duty) and therefore, students could not stay in the classroom.

Consequently, this particularity allowed us to conduct a natural experiment (i.e., quasi-experiment) to examine the relationship between using recess time for other purposes and children’s PA levels. Hence, in this quasi-experimental design, the “B” group was the group of students that used the full time of recess freely and the “A” group was the group of students that used some or all part of the time of recess for other purposes. Likewise, recess time on Fridays was used as control day to conduct nonequivalent comparison group analyses. Hence, this natural experiment cannot be intentionally replicated. In this regard, ethics was gained because the procedure of data collection was not modified. As aforementioned, all students followed their normal daily school routines, with PA being monitored during the complete school day. Only the data analysis was adapted to this particularity that required a comparison between the two groups.

### Data Analysis

All data were analyzed using IBM SPSS Statistics 24.0 software (IBM, Chicago, IL). Descriptive analysis of the time spent in sedentary behavior, light PA, moderate PA, vigorous PA, and MVPA during school recess were executed. This study aimed to examine the behavior of two response variables along a specific period (23 school recesses), which generates a certain dependence between the observations of the same subject or cluster. Therefore, Generalized Estimating Equations (GEE; Liang & Zeger, 1986) were used because this data analysis technique considers such association. The association structure is incorporated into the process of estimating the average response which allows the researchers to obtain more accurate estimates and to execute accurate statistical analysis (Stokes et al., 2000). Furthermore, using the GEE method allows for observations to be examined with different sample sizes or when there is missing data which cannot be done by the method of weighted least squares (Stokes et al., 2000). Since the response variables (i.e., METs and sedentary behavior) follow a normal distribution, a GEE model was performed following Liang and Zeger (1986). Therefore, two GEE models were used to analyze both the repeated observations of the METs (i.e., dependent variable) and the repeated observations of the sedentary behavior in which the effects of the variables gender, group, day of the week, week, BMI, and academic achievement were specified.

## Results

### Preliminary Analysis

Table 1 shows the average METs and sedentary time yielded during school recess according to gender, age, BMI, and group to which students belonged (i.e., A or B). Likewise, average METs and sedentary time yielded on Fridays (i.e., control day) are represented for both groups.

**Table 1:** Average METs and sedentary time (minutes) yielded during school recess according to gender, age, body mass index (BMI) and group to which students belonged; and results from control day (i.e., Fridays)

	*N*	METs *M* (*SD*)	Sedentary behavior *M* (*SD*)
Overall	46	3.79 (1.04)	7.98 (5.48)
Gender			
Boys	19	3.84 (1.05)	7.94 (5.89)
Girls	27	3.76 (1.02)	8.02 (5.18)
Age			
5	8	3.08 (0.84)	9.78 (6.48)
6	38	4.10 (1.20)	7.16 (6.89)
Group			
A	23	3.50 (1.04)	9.52 (6.16)
B*	23	4.14 (0.93)	6.14 (3.77)
Control day			
A*	23	4.02 (0.99)	6.37 (4.26)
B*	23	3.90 (0.93)	6.98 (3.59)
BMI			
Underweight	3	3.58 (0.97)	8.57 (5.86)
Normal/healthy	36	3.88 (1.04)	7.59 (5.94)
Overweight/obese	7	3.43 (0.95)	9.73 (6.15)

*No child was deprived of recess at any time

Students spent 60.8 ± 19.2% of school recess in MVPA (62.01% boys and 59.6% girls) and 26.6 ± 18.26% of school recess in sedentary behavior (26.46 ± 19.63% boys and 27.73 ± 17.73% girls). The different periods they were involved in the different PA intensities are shown in Table 2. It can be observed that after the 23 recess periods recorded, students from the “A” group spent more time in sedentary behavior and less time in moderate and vigorous PA than students from the “B” group.

**Table 2:** Time (minutes) and percentages in sedentary behavior, light physical activity (PA), moderate PA, vigorous PA, and moderate vigorous PA (MVPA) during school recess

PA intensity	Overall total time	Overall percentage	A Group		B Group	
	M (SD)	%	M (SD)	%	M (SD)	%
Sedentary behavior	7.98 (5.48)	26.6	9.52 (6.16)	31.7	6.14 (3.77)	20.46
Light PA	3.78 (1.54)	12.6	3.78 (1.51)	12.6	3.77 (1.58)	12.56
Moderate PA	15.47 (4.68)	51.56	14.51 (5.26)	48.36	16.62 (3.55)	55.4
Vigorous PA	2.77 (2.54)	9.23	2.18 (2.17)	7.26	3.47 (2.76)	11.56
MVPA	18.24 (5.76)	60.8	16.7 (6.28)	55.66	20.09 (4.41)	66.95

Elementary school recess lasted 30 min. Means were calculated from the 23 recess periods

### Generalized Estimating Equations Analysis

**Fig. 1:**
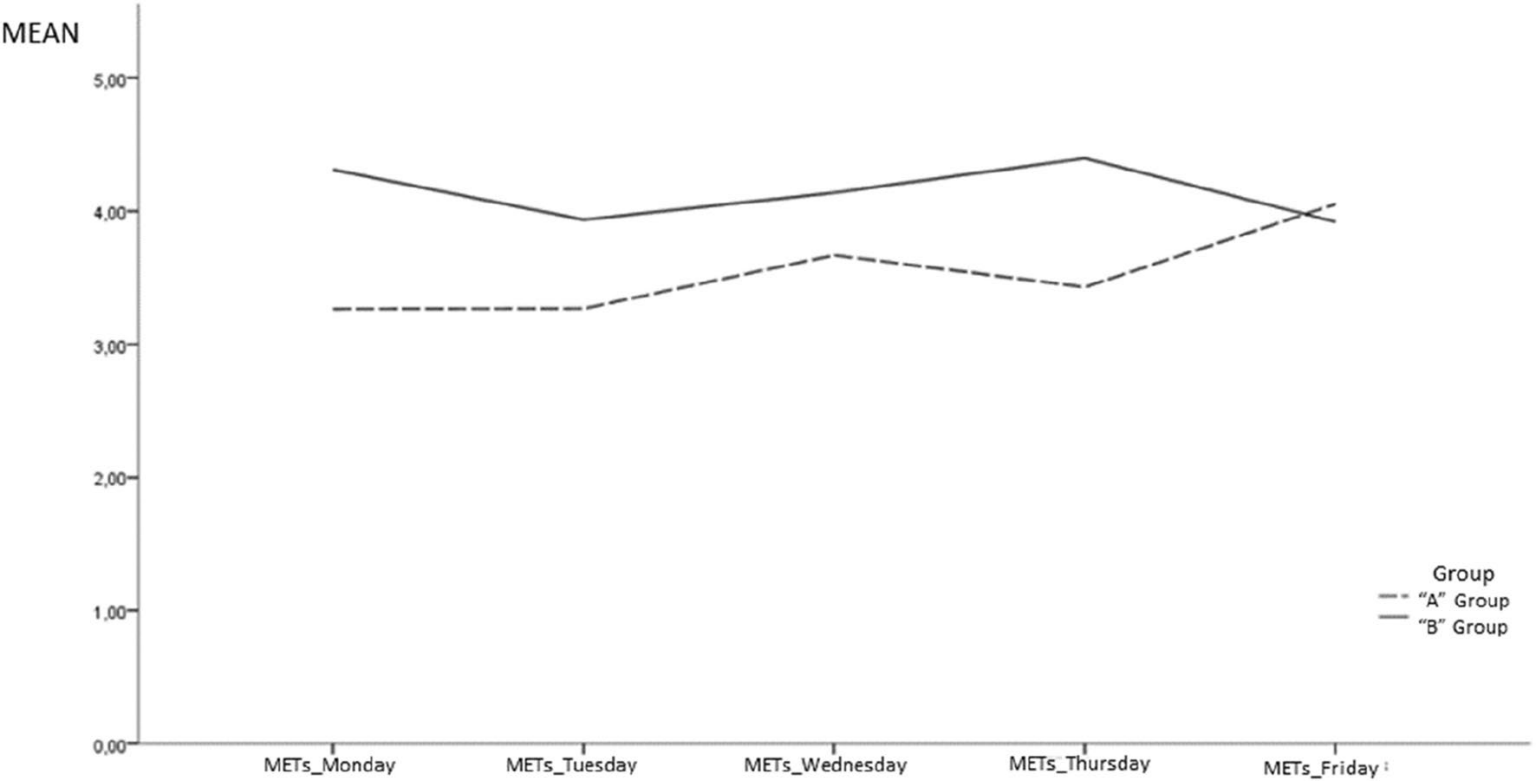
Different patterns of METs observed across the week

The repeated measures of METs in school recess show that there is a significant relationship between the PA intensity and the following variables (see Table 3): gender, (boys scored higher than girls); BMI (children within a normal or healthy range had higher levels than overweight/obese children and underweight children); day of the week (students yielded the highest METs on Friday and the second-highest on Thursdays); weeks (there are significant differences between the 6th week in regard to the 4th and the 5th week); groups (students from the “B” group yielded higher METs than the students from the “A” group. Figure 1 shows the different patterns of METs observed between both groups); and finally, academic achievement showed a positive relationship with the METs yielded during school recess. On the other side, age (measured in months) was not related to the METs.

**Table 3:** Generalized estimating equation model predicting METS and sedentary behavior

Factor	METs			Sedentary behavior		
	OR	95% CI	*p*-Value	OR	95% CI	*p*-value
Gender						
Boys	1.55	1.34–1.80	.000	0.20	0.09–0.45	.000
Girls	1.00			1.00		
BMI						
Underweight	0.74	0.54–0.99	.048	2.11	0.37–11.9	.397
Normal/healthy	1.28	1.08–1.53	.000	0.34	0.11–1.02	.054
Overweight/obese	1.00			1.00		
Day						
Monday	0.68	0.55–0.84	.000	9.25	3.37–25.42	.000
Tuesday	0.56	0.45–0.71	.000	35.47	11.61–108.33	.000
Wednesday	0.77	0.61–0.97	.025	8.72	2.78–27.38	.000
Thursday	0.84	0.68–1.04	.112	4.77	1.73–13.71	.003
Friday	1.00			1.00		
Week						
1st	0.88	0.66–1.17	.376	2.95	0.59–14.8	.188
2nd	0.96	0.73–1.27	.774	1.88	0.42–8.42	.410
3rd	0.80	0.61–1.06	.125	2.47	0.54–11.2	.241
4th	0.72	0.54–0.95	.020	5.09	1.08–24.0	.040
5th	1.42	1.05–1.91	.022	0.12	0.02–0.52	.005
6th	1.00			1.00		
Group						
B	2.15	1.89–2.45	.000	0.02	0.01–0.04	.000
A	1.00			1.00		
Age	0.99	0.97–1.01	.127	1.07	0.96–1.19	.193
Academic achievement	1.11	1.05–1.17	.000	0.65	0.48–0.87	.004

Regarding the repeated measures of sedentary behavior during school recess, it can be also observed in Table 3 that there is a significant relationship between sedentary behavior and the following variables: gender (girls scored higher than boys); day of the week (students spend less time in sedentary behavior on Fridays than on other days of the week); weeks (there are significant differences between the 6th week in regards to the 4th and the 5th weeks); groups (students from the “A” group spend more time in sedentary behavior than the students from the “B” group); and finally, the academic achievement showed negative associations with the sedentary behavior spent during school recess. On the other side, age (measured in months) and BMI were not related to sedentary behavior during school recess.

## Discussion

One purpose of this study was to assess first-grade students’ PA levels and sedentary behavior during school-recess. The results of this study showed that first-grade students spent a large part of recess in MVPA (60.8%) and also a significant percentage in sedentary behavior (18.26%). Specifically, boys participated in more MVPA and less sedentary behavior during recess time than girls, which is consistent with previous studies that have analyzed both PA (Ridgers et al., 2006, 2010) and sedentary behavior (Greca, 2017; Ridgers et al., 2010) in children during school recess. These differences have been explained by social factors.

The analysis of PA during recess showed that BMI was not associated with sedentary behavior but it was related to METs, with normal-weight children achieving higher METs than those children who were underweight and overweight/obese, respectively. These results are consistent with recent studies that found that overweight children performed more moderate intensity PA and less vigorous intensity PA than non-overweight children during school recess (Ridgers et al., 2014; Stratton et al., 2007). The reasons for these relationships are not widely known, but it has been suggested that overweight children might not be able to engage at higher intensities due to fitness or low movement skills (Ridgers et al., 2014).

It was observed that METs were positively associated with the academic achievement of students, and also that sedentary behavior was negatively associated with academic achievement. In other words, in this sample, children who get more involved in more intense PA during recess achieved higher academic grades than those that yielded fewer METs and than those that spent more time participating in sedentary behavior. There is inadequate evidence to conclude that increased PA in school may enhance academic achievement in all children (Resaland et al., 2016). However, these findings support the idea that PA is beneficially associated with cognitive performances (Ellemberg & St-Lous-Deschênes, 2010; Fedewa & Ahn, 2011), learning processes (Graham et al., 2014; Mullender-Wijnsma et al., 2016), and children’s academic achievements (Gonzalez-Sicilia et al., 2019; Xu et al., 2017; Zeng et al., 2017), which have been explained through different hypotheses.

For instance, one approach is that PA may enhance arousal and minimize fatigue and boredom (Shepard, 1996). Other neurological perspectives state that longer periods of regular MVPA lead to changes in brain structure, neurotransmitter concentration, and function which may improve cognitive functions such as attention, concentration, and working memory (Donnelly et al., 2016; Hillman et al., 2008). Although the results of this study could be explained through these perspectives, more research is needed to investigate the impact of PA during school recess on children’s academic achievement.

The literature shows contradictory results regarding the relationships between sedentary behavior and academic achievement. For instance, some studies have found that the time that children spend in sedentary behavior is either, not associated (Lopes et al., 2017), or negatively associated with their academic achievement (Tremblay et al., 2011). However, such relationships were established primarily through the time spent in screen time (e.g., TV viewing, computer use, videogames) and not during school recess time. Therefore, the results of this study would be more consistent with recent studies that found unfavorable associations between long sedentary periods and low MVPA at school with academic achievement (Mooses et al., 2017).

The other aim of this study was to investigate the relationship between withholding school recess for academic or disciplinary reasons and children’s PA levels. A natural experiment was conducted due to the different methodological approaches observed between the two teachers in regards to their use of the school recess time. The teacher from the “A” group had to supervise students during recess time on Fridays; as a result, students could not stay in the classroom. Preliminary descriptive analysis showed similar patterns on the METs and on the sedentary behavior time yielded on Fridays between the “A” group and the “B” group. However, the GEE model for repeated measures revealed that the METs yielded on Fridays were significantly higher than on the other days of the week (except Thursdays that show non-significant differences) and, moreover, that students from the “B” group yielded higher METs during school recess than the students from the “A” group. The same pattern was observed regarding sedentary behavior, with significantly lower periods of sedentary behavior on Fridays than on the other days of the week and with significantly more time spent in sedentary behavior among students form the “A” group when compared with the students from the “B” group. This would mean that students from the “A” group generally yielded lower PA intensities during the school recess than the students from the “B” group, but even more concerning is the fact that regarding sedentary behavior, the differences are more noticeable between the groups.

Recess provides a daily opportunity for students to be physically active. Moreover, there is literature suggesting that children improve their class behavior and attention after recess (Ramstetter et al., 2010). However, several studies have reported that in some instances children’s poor classroom behavior is punished with reduced time for recess (Huberty et al., 2012; Ramstetter et al., 2010; Turner et al., 2013). Thus, this study showed that the teacher of one group tended to use all or part of recess for other purposes; and this practice had a negative impact on children’s PA levels and extended their sedentary behavior. Several works have observed that participation in recommended levels of PA promotes children’s health (Penedo & Dahn, 2005) and prevents childhood obesity (Janssen & LeBlanc, 2010). Likewise, Xu et al. (2017) observed that an increase in PA during the morning time had positive benefits for students throughout the school day (e.g., increased scores on math standard score or greater confidence in their academic ability). Consequently, since school recess might contribute to more than 40% of recommended daily PA levels (Ridgers et al., 2006), it should be considered as a unique occasion to optimize not only physical but emotional, social, and cognitive development (Ramstetter et al., 2010).

To date, no studies have empirically examined the impact of using school recess for other purposes (e.g., to punish children misbehavior or to finish class work) on the PA and sedentary behavior of children who are 6-years-old. This kind of study cannot be intentionally replicated because of the ethical implications. Therefore, this might be the most important finding of this study. On the other hand, this study is consistent with studies that found positive relationships between academic achievement and different forms of PA such as leisure-time PA (González Sicilia et al., 2019), physical education, sports (Howie & Pate, 2012), or intervention programs at school (e.g., Layne et al., 2021). Thus, this study also shows the positive relationship between PA intensity during recess time (i.e., an unstructured PA) and academic achievement in a natural setting with 6-year-old children.

## Limitations and Future Research

Though the results provided in this and previous studies contribute to highlighting the importance of school recess for children, this research acknowledges some limitations. First, the sample size is small and only involved a convenience sample of first-year students. Second, PA levels only were measured by accelerometry and observational data could have enhanced the measurements (e.g., what was the daily pattern of students?). Moreover, all measures were collected over 6 weeks in the fall and PA patterns might be different during other times of the academic year, with other teachers or in other regions or countries. Thus, the longitudinal design of this study and the geographic location of the study are also potential limitations to generalizability.

Finally, the relationships between PA and academic achievement should be taken cautiously because other confounding factors should be taken into consideration, such as socioeconomic variables or the PA that is undertaken outside of recess/school hours. Therefore, further research should evaluate the PA levels during school recess with other age students, in longer periods, with other data collection strategies, and considering other potential confounding variables. However, this study shows interesting findings that allow us to better understand the current situation of school-age students regarding school recess, and contribute to reflect on the importance of school recess and the impact of withholding recess to punish children misbehavior or to finish classwork at the cost of children’s time for being physically active.

## Conclusion

In conclusion, the main finding of this study is that it has been empirically observed that using some part of the recess to work on other educational issues is related with children’s sedentary behavior and PA. Hence, the data presented supports all those arguments that have led to actively discouraging schools from practices that exclude students from all or part of recess and encourage teachers to avoid such practices because school recess plays an important role in school-age students’ PA and students should not be excluded from participation in all or part of recess for punitive or academic reasons. Many teachers often believe that withholding recess from students is an effective tool for punishing bad behavior, however, they should take into account that such practice is counterproductive, especially for children that really need a break. Thus, instead of “discouraging” from taking away a student’s recess time, stronger policies should either limit or prohibit a teacher or administrator from withholding recess for punitive or academic issues. School policies should provide teachers training or instruction in strategies more effective than simply taking away recess, and teachers should only be able to withhold recess for specific reasons.

## References

[CR1] Ainsworth BE, Haskell WL, Herrmann SD, Meckes N, Bassett DR, Tudor-Locke C (2011). Compendium of physical activities: A second update of codes and MET values. Medicine and Science in Sports and Exercise.

[CR2] Barnett WS, Jung K, Yarosz DJ, Thomas J, Hornbeck A, Stechuk R, Burns S (2008). Educational effects of the tools of the mind curriculum: A randomized trial. Early Childhood Research Quarterly.

[CR3] Biddle SJ, Bennie JA, Bauman AE, Chau JY, Dunstan D, Owen N (2016). Too much sitting and all-cause mortality: Is there a causal link?. BMC Public Health.

[CR4] Bull, F.C., Al-Ansari, S.S., Biddle, S., Borodulin, K., Buman, M.P., Cardon, G., ... Willumsen, J.F. (2020). World Health Organization 2020 guidelines on physical activity and sedentary behaviour. *British Journal of Sports Medicine*, *54*(24), 1451–1462.10.1136/bjsports-2020-102955 10.1136/bjsports-2020-102955 PMC7719906 33239350

[CR5] Cardon G, De Clercq D, De Bourdeaudhuij I, Breithecker D (2004). Sitting habits in elementary schoolchildren: A traditional versus a “Moving school”. Patient Education and Counseling.

[CR6] Carriedo A, Cecchini JA, Fernández-Río J, Méndez-Giménez A (2020). Resilience and physical activity in people under home isolation due to COVID-19: A preliminary evaluation. Mental Health and Physical Activity.

[CR7] Carson V, Hunter S, Kuzik N, Gray CE, Poitras VJ, Chaput JP (2016). Systematic review of sedentary behaviour and health indicators in school-aged children and youth: An update 1. Applied Physiology, Nutrition, and Metabolism.

[CR8] Cecchini JA, Carriedo A (2020). Effects of an interdisciplinary approach integrating mathematics and physical education on mathematical learning and physical activity levels. Journal of Teaching in Physical Education.

[CR9] Centers for Disease Control Prevention: CDC. (2015). *About child & teen BMI*: May 15, 2015. Retrieved June 14, 2017 from https://www.cdc.gov/healthyweight/assessing/bmi/childrens_bmi/about_childrens_bmi.html

[CR10] Cliff DP, Jones RA, Burrows TL, Morgan PJ, Collins CE, Baur LA, Okely AD (2014). Volumes and bouts of sedentary behavior and physical activity: Associations with cardiometabolic health in obese children. Obesity.

[CR11] Cooper, A. R., Goodman, A., Page, A. S., Sherar, L. B., Esliger, D. W., van Sluijs, E. M., ... Ekelund, U. (2015). Objectively measured physical activity and sedentary time in youth: The International children’s accelerometry database (ICAD). *International Journal of Behavioral Nutrition and Physical Activity*, *12*(1), 1–10. 10.1186/s12966-015-0274-5 10.1186/s12966-015-0274-5 PMC4574095 26377803

[CR12] de Greeff JW, Bosker RJ, Oosterlaan J, Visscher C, Hartman E (2018). Effects of physical activity on executive functions, attention and academic performance in preadolescent children: A meta-analysis. Journal of Science and Medicine in Sport.

[CR13] Donnelly JE, Hillman CH, Castelli D, Etnier JL, Lee S, Tomporowski P (2016). Physical activity, fitness, cognitive function, and academic achievement in children: A systematic review. Medicine & Science in Sports & Exercise.

[CR14] Dunton GF, Do B, Wang SD (2020). Early effects of the COVID-19 pandemic on physical activity and sedentary behavior in children living in the US. BMC Public Health.

[CR15] Ellemberg D, St-Louis-Deschênes M (2010). The effect of acute physical exercise on cognitive function during development. Psychology of Sport and Exercise.

[CR16] Erickson, K.I., Hillman, C., Stillman, C.M., Ballard, R.M., Bloodgood, B., Conroy, D E., ... Powell, K. E. (2019). Physical activity, cognition, and brain outcomes: A review of the 2018 physical activity guidelines. *Medicine and Science in Sports and Exercise*, *51*(6), 1242. 10.1249/MSS.0000000000001936 10.1249/MSS.0000000000001936 PMC6527141 31095081

[CR17] Fedewa AL, Ahn S (2011). The effects of physical activity and physical fitness on children’s achievement and cognitive outcomes: A meta-analysis. Research Quarterly for Exercise and Sport.

[CR18] Freedson P, Pober D, Janz KF (2005). Calibration of accelerometer output for children. Medicine & Science in Sports & Exercise.

[CR19] Gonzalez-Sicilia D, Brière FN, Pagani LS (2019). Prospective associations between participation in leisure-time physical activity at age 6 and academic performance at age 12. Preventive Medicine.

[CR20] Graham DJ, Lucas-Thompson RG, O’Donnell MB (2014). Jump in! An investigation of school physical activity climate, and a pilot study assessing the acceptability and feasibility of a novel tool to increase activity during learning. Frontiers in Public Health.

[CR21] Greca J (2017). Sedentary behavior during school recces in southern Brazil. Perceptual and Motor Skill.

[CR22] Hillman CH, Erickson KI, Kramer AF (2008). Be smart, exercise your heart: Exercise effects on brain and cognition. Nature Reviews Neuroscience.

[CR23] Howie EK, Pate RR (2012). Physical activity and academic achievement in children: A historical perspective. Journal of Sport and Health Science.

[CR24] Huberty J, Dinkel D, Coleman J, Beighle A, Apenteng B (2012). The role of schools in children’s physical activity participation: Staff perceptions. Health Education Research.

[CR25] Janssen I, LeBlanc AG (2010). Systematic review of the health benefits of physical activity and fitness in school-aged children and youth. International Journal of Behavioral Nutrition and Physical Activity.

[CR26] Júdice PB, Silva AM, Berria J, Petroski EL, Ekelund U, Sardinha LB (2017). Sedentary patterns, physical activity and health-related physical fitness in youth: A cross-sectional study. International Journal of Behavioral Nutrition and Physical Activity.

[CR27] Kim Y, Beets MW, Welk GJ (2012). Everything you wanted to know about selecting the “right” Actigraph accelerometer cut-points for youth, but…: A systematic review. Journal of Science and Medicine in Sport.

[CR28] Layne T, Yli-Piipari S, Knox T (2021). Physical activity break program to improve elementary students’ executive function and mathematics performance. Education 3–13.

[CR29] Liang KY, Zeger SL (1986). Longitudinal data analysis using generalized linear models. Biometrika.

[CR30] Lopes L, Santos R, Mota J, Pereira B, Lopes V (2017). Objectively measured sedentary time and academic achievement in schoolchildren. Journal of Sports Sciences.

[CR31] Marques A, Minderico C, Martins S, Palmeira A, Ekelund U, Sardinha LB (2016). Cross-sectional and prospective associations between moderate to vigorous physical activity and sedentary time with adiposity in children. International Journal of Obesity (Lond).

[CR32] Mooses K, Mägi K, Riso EM, Kalma M, Kaasik P, Kull M (2017). Objectively measured sedentary behaviour and moderate and vigorous physical activity in different school subjects: A cross-sectional study. BMC Public Health.

[CR33] Mullender-Wijnsma MJ, Hartman E, de Greeff JW, Doolaard S, Bosker RJ, Visscher C (2016). Physically active math and language lessons improve academic achievement: A cluster randomized controlled trial. Pediatrics.

[CR34] Page A, Cooper A, Stamatakis E, Foster L, Crowne E, Sabin M (2005). Physical activity patterns in nonobese and obese children assessed using minute-by-minute accelerometry. International Journal of Obesity.

[CR35] Penedo FJ, Dahn JR (2005). Exercise and well-being: A review of mental and physical health benefits associated with physical activity. Current Opinion in Psychiatry.

[CR36] Poitras, V. J., Gray, C. E., Borghese, M. M., Carson, V., Chaput, J. P., Janssen, I., ... Tremblay, M. S. (2016). Systematic review of the relationships between objectively measured physical activity and health indicators in school-aged children and youth. *Applied Physiology, Nutrition, and Metabolism*, *41*(6), S197–S239.10.1139/apnm-2015-0663 10.1139/apnm-2015-0663 27306431

[CR37] Ramstetter CL, Murray R, Garner AS (2010). The crucial role of recess in schools. Journal of School Health.

[CR38] Resaland GK, Aadland E, Moe VF, Aadland KN, Skrede T, Stavnsbo M (2016). Effects of physical activity on schoolchildren’s academic performance: The Active Smarter Kids (ASK) cluster-randomized controlled trial. Preventive Medicine.

[CR39] Ridgers ND, Fairclough SJ, Stratton G (2010). Variables associated with children’s physical activity levels during recess: The A-CLASS project. International Journal of Behavioral Nutrition and Physical Activity.

[CR40] Ridgers ND, Saint-Maurice PF, Welk GJ, Siahpush M, Huberty JL (2014). Non-overweight and overweight children’s physical activity during school recess. Health Education Journal.

[CR41] Ridgers ND, Stratton G, Fairclough SJ (2006). Physical activity levels of children during school playtime. Sports Medicine.

[CR42] Saint-Maurice PF, Kim Y, Welk GJ, Gaesser GA (2016). Kids are not little adults: What MET threshold captures sedentary behavior in children?. European Journal of Applied Physiology.

[CR43] Sedentary Behaviour Research Network (2012). Letter to the editor: Standardized Use of the terms “sedentary” and “sedentary behaviours”. Applied Physiology, Nutrition, and Metabolism.

[CR44] Shepard RJ (1996). Habitual physical activity and academic performance. Nutritional Review.

[CR45] Stokes ME, David CS, Koch GG (2000). Categorical data analysis using the SAS system.

[CR46] Stratton G, Ridgers ND, Fairclough SJ, Richardson DJ (2007). Physical activity levels of normal-weight and overweight girls and boys during primary school recess. Obesity (Silver Spring).

[CR47] Strong WB, Malina RM, Blimke CJ, Daniels SR, Dishman RK, Gutin B (2005). Evidence based physical activity for school-age youth. Journal of Pediatrics.

[CR49] Tremblay MS, LeBlanc AG, Kho ME, Saunders TJ, Larouche R, Colley RC (2011). Systematic review of sedentary behavior and health indicators in school-aged children and youth. International Journal of Behavioral Nutrition and Physical Activity.

[CR50] Turner L, Chriqui JF, Chaloupka FJ (2013). Withholding recess from elementary school students: Policies matter. Journal of School Health.

[CR51] World Health Organization. (2004). *Global strategy on diet, physical activity and health*. Geneva: WHO Press. Available at http://www.who.int/dietphysicalactivity/goals/en/index.html.

[CR52] Xu T, Byker EJ, Gonzales MR (2017). Ready to learn: The impact of the Morning Blast physical activity intervention on elementary school students. Movement, Health & Exercise.

[CR54] Zeng N, Ayyub M, Sun H, Wen X, Xiang P, Gao Z (2017). Effects of physical activity on motor skills and cognitive development in early childhood: A systematic review. BioMed Research International.

[CR55] Zhang G, Wu L, Zhou L, Lu W, Mao C (2016). Television watching and risk of childhood obesity: A meta-analysis. European Journal of Public Health.

[CR56] Zimmo L, Farooq A, Almudahka F, Ibrahim I, Al-Kuwari MG (2017). School-time physical activity among Arab elementary school children in Qatar. BMC Pediatrics.

